# A Naturally Derived Watercress Flower-Based Phenethyl Isothiocyanate-Enriched Extract Induces the Activation of Intrinsic Apoptosis via Subcellular Ultrastructural and Ca^2+^ Efflux Alterations in an In Vitro Model of Human Malignant Melanoma

**DOI:** 10.3390/nu15184044

**Published:** 2023-09-18

**Authors:** Sotiris Kyriakou, Louiza Potamiti, Nikoletta Demosthenous, Tom Amery, Kyle Stewart, Paul G. Winyard, Rodrigo Franco, Aglaia Pappa, Mihalis I. Panayiotidis

**Affiliations:** 1 Department of Cancer Genetics, Therapeutics & Ultrastructural Pathology, The Cyprus Institute of Neurology & Genetics, Nicosia 2371, Cyprus; sotirisk@cing.ac.cy (S.K.); louizap@cing.ac.cy (L.P.); nicolettad@cing.ac.cy (N.D.); 2 The Watercress Company, Dorchester DT2 8QY, UK; tom.amery@thewatercresscompany.com; 3 Watercress Research Limited, Exeter EX5 2GE, UK; kyle@watercress-research.com (K.S.); paul@watercress-research.com (P.G.W.); 4 Redox Biology Centre, University of Nebraska, Lincoln, NE 68583, USA; rodrigo.franco@unl.edu; 5 Department of Veterinary Medicine & Biomedical Sciences, University of Nebraska, Lincoln, NE 68583, USA; 6 Department of Molecular Biology & Genetics, Democritus University of Thrace, 68100 Alexandroupolis, Greece; apappa@mbg.duth.gr

**Keywords:** watercress, phenethyl isothiocyanate, mitochondria, endoplasmic reticulum, calcium efflux, apoptosis

## Abstract

The aim of the current study was to (i) extract isolated fractions of watercress flowers enriched in polyphenols, phenethyl isothiocyanate and glucosinolates and (ii) characterize the anticancer mode of action of non-lethal, sub-lethal and lethal concentrations of the most potent extract fraction in primary (A375) and metastatic (COLO-679) melanoma cells as well as non-tumorigenic immortalized keratinocyte (HaCaT) cells. Cytotoxicity was assessed via the Alamar Blue assay, whereas ultrastructural alterations in mitochondria and the endoplasmic reticulum were determined via transmission electron microscopy. Mitochondrial membrane depolarization was determined using Mito-MP dye, whereas apoptosis was evaluated through the activation of caspases-3, -8 and -9. Among all extract fractions, the phenethyl isothiocyanate-enriched one (PhEF) possessed significant cytotoxicity against A375 and COLO-679 cells, while HaCaT cells remained relatively resistant at sub-lethal and lethal concentrations. Additionally, ultrastructural subcellular alterations associated with apoptosis were observed by means of increased mitochondrial area and perimeter, decreased cristae density and a shorter distance of the endoplasmic reticulum to the mitochondria, all taking place during “early” time points (2–4 h) of exposure. Moreover, PhEF induced mitochondrial membrane depolarization associated with “late” time points (24 h) of exposure, thereby leading to the activation of intrinsic apoptosis. Finally, the inhibition of cytosolic Ca^2+^ efflux reduced levels of caspases-9 and -3 activity, suggesting the involvement of Ca^2+^ efflux in modulating the activation of intrinsic apoptosis. To conclude, our data demonstrate an association of “early” ultrastructural alterations in mitochondria and the endoplasmic reticulum with the “late” induction of intrinsic apoptosis via the modulation of Ca^2+^ efflux.

## 1. Introduction

Watercress (*Nasturtium officinale*) is an aquatic cruciferous plant of the *Brassicaceae* family (which includes cauliflower, horseradish, cabbage, broccoli and wasabi) with a well-documented nutritional benefit. In various epidemiological studies, the positive correlation between supplementation with watercress and risk reduction for various diseases has been emphasized [1,2]. For example, various studies have documented its use against hyperglycemia, hypertension, hypercholesterolemia, odontalgia, scurvy, bronchitis, diuresis and arthritis [3,4,5,6,7,8]. In addition, the anticancer potency of watercress has been intensively studied by various groups [9,10,11,12] and was shown to rely on its high content of (i) aliphatic, indolyl and aromatic isothiocyanates (ITCs; secondary metabolites yielded from the hydrolysis of their precursor molecules (glucosinolates) via the action of myrosinase) and (ii) various polyphenolic compounds (e.g., quercetin-3-*O*-rutinoside, *p*-coumaric and ferulic acid) [13,14,15,16,17]. Specifically, our group has shown that watercress is an enriched source of phenethyl isothiocyanate (PEITC) (273.89 ± 0.88 ng/g of dry extract), indole-3-carbinol (191.44 ± 1.99 ng/g of dry extract), quercetin (1459.30 ± 12.95 ng/g of dry extract)- and kaempferol-3-*O*-rutinosides (257.54 ± 2.31 ng/g of dry extract), isorhamnetin (289.40 ± 1.37 ng/g of dry extract), protocatechuic (134.72 ± 1.21 ng/g of dry extract) and chlorogenic (41.34 ± 1.00 ng/g of dry extract) acids, among others [18,19].

Recently, a comprehensive chemical characterization of the aerial parts of watercress (including leaves, stems and flowers) revealed an increased accumulation of phytochemicals (phenolic acids, ITCs and glucosinolates) in watercress flowers followed by those in leaves and stems [18]. Furthermore, it was concluded that these elevated ITCs levels, in watercress flowers, do not entirely serve as a defense mechanism, but they also act as pollinator signal attractors related to plant reproduction [20,21,22]. Finally, the high content of these phytochemicals in watercress flowers allows the opportunity for further exploitation of this part of the plant, thereby targeting its potential anticancer capacity.

More specifically, Boyd et al. (2006) have documented, for the first time, that watercress extract can act as an anticancer agent through the inhibition of the proliferation and metastasis of HT-29 colon cancer cells through suppressing oxidative DNA damage [23,24]. In another case, it was demonstrated that that the metastatic and invasiveness capacity of MDA-MB-23 breast cancer cells was inhibited upon exposure to watercress extracts [25]. This effect was attributed to the high levels of lipid peroxidation inhibitors, metal chelators and reducing agents within the isolated extract, all of which can act synergistically and/or additively [25,26,27]. To this end, it was previously reported that PEITC acts as a cytotoxic agent against prostate, leukemia, colon, liver, cervical, lung, multiple myeloma and malignant melanoma [28,29,30,31,32,33].

According to various research studies, PEITC can effectively target a wide spectrum of cancer-regulated proteins [34,35]. To this end, Zigang et al. suggested that PEITC can activate apoptotic cascades in mouse epidermal JB6 cells [36]. Specifically, in doing so, the cytotoxic range of PEITC concentrations was between 120 nM to 4 μM [36], thereby suggesting similarities with the utilization of various chemotherapeutic drugs including daunomycin (4 μΜ) [37] and itraconazole (5 μM) in human breast carcinoma cells [38]. These results imply PEITC’s anticancer potential is comparable to the action of various conventional clinical chemotherapeutic drugs [38]. Consequently, it is evidenced that the utilization of plant extracts can have a comparable therapeutic index as to that of conventional clinical drugs, perhaps suggesting their utilization as adjuvants in combinatorial therapeutic protocols aiming to reduce the potential toxic side effects induced by the action of clinically used drugs [39].

Therefore, the aim of this study was twofold: (i) to characterize the cytotoxic potency of various watercress flower-based extract fractions enriched with either PEITC (PhEF), polyphenols (PoEF) or glucosinolates (GluEF) and (ii) to further delineate the underlined mechanism(s) of the anticancer mode of action of non-lethal, sub-lethal and lethal concentrations of the most potent fraction in an in vitro model of human malignant melanoma consisting of human primary (A375) and metastatic (COLO-679) malignant melanoma as well as non-tumorigenic immortalized keratinocyte (HaCaT) cells.

## 2. Materials and Methods

Solvents: water (HPLC grade), methanol (LC-MS grade, purity ≥ 99.9%), petroleum ether (HPLC grade, purity ≥ 99.9) and acetone (HPLC grade, purity ≥ 99.8%) were all purchased from Medisell Co., Ltd., Nicosia, Cyprus. Reagents: dimethyl sulfoxide (DMSO) (PanBiotech, Athens, Greece), resazurin sodium salt (Fluorochem, Derbyshire, UK), carbonyl cyanide *m*-chlorophenyl hydrazine (CCCP) (purity ≥ 97%, Sigma Aldrich, Nicosia, Cyprus), *tert*-butyl hydroperoxide (TBH) (70% in water, Sigma Aldrich, Nicosia, Cyprus), 1,2-bis(2-aminophenoxy)ethane-*N*,*N*,*N′*,*N′*-tetraacetic acid tetrakis (acetoxymethyl ester) (BAPTA-AM) (purity ≥ 95%, Sigma Aldrich, Nicosia, Cyprus), glutaraldehyde (25%, Agar Scientific, Essex, UK), osmium tetroxide (Agar Scientific, Essex, UK), propylene oxide (Agar Scientific, Essex, UK), araldite resin (Agar Scientific, Essex, UK), agar 100 resin (Agar Scientific, Essex, UK), DDSA (Agar Scientific, Essex, UK), DMP-30 (Agar Scientific, Essex, UK), uranyl acetate (Agar Scientific, Essex, UK) and lead citrate (Agar Scientific, Essex, UK). Fluorescence mounting medium was purchased from (Dako-Agilent, Santa Clara, CA, USA, and *para*-formaldehyde was from Sigma Aldrich, Nicosia, Cyprus. Assay kits: Bicinchoninic acid (BCA) protein assay kit (Thermo Scientific, Waltham, MA, USA), MT-1 MitoMP detection kit (Dojindo, Munich, Germany) and a caspase-3, -8 and -9 multiplex activity fluorometric assay kit (Abcam, Cambridge, UK). Cell culture reagents: Dulbecco’s Modified Eagles Medium (DMEM) high-glucose media, Roswell Park Memorial Institute (RPMI) 1640, fetal bovine serum (FBS), pen/strep (100 U/mL penicillin, 100 μg/mL streptomycin), *L*-glutamine, trypsin-EDTA (100×) and phosphate-buffered saline (PBS) *w/o* calcium and *w/o* magnesium were all purchased from BIOSERA, Athens, Greece.

### 2.1. Plant Material Cultivation, Processing and Storage

Fresh watercress flower samples were kindly provided by The Watercress Company, Dorchester, UK. The flowers were kept at −20 °C in a sealed bag protected from humidity and light until further use. Liquid nitrogen was sprayed into watercress flowers and then the frozen material was dried in a freeze-drier (Chris Alpha 1-4, LCS Basis) at −55 °C and 0.05 mbar for 48 h. The de-hydrated parts were immersed in liquid nitrogen, pulverized via a domestic blender and stored at −20 °C in a sealed bag in order to avoid any exposure to either light or humidity or air.

### 2.2. Extraction of Phenethyl Isothiocyanate Content

The extraction protocol was performed as previously published [18,19,33]. Briefly, five (5.0) grams of the powdered flower watercress sample was mixed with a catalytic amount of ascorbic acid in 315 mL of phosphate-buffered saline (PBS), and the resulting suspension was stirred at 37 °C for 2 h, in order to promote the hydrolysis of its precursor glucosinolate, gluconasturtiin. The extraction of phenethyl isothiocyanate was achieved via liquid–liquid extraction utilizing *n*-hexane (400 mL). The organic phase was isolated, dried over magnesium sulphate and concentrated to dryness, producing the phenethyl isothiocyanate-enriched extract as a viscous oil.

### 2.3. Extraction of Polyphenols’ Content

The extraction protocol was performed as previously published [18,19,33]. Five (5.0) grams of the powdered flower watercress sample was mixed with 80% aqueous methanol. The resulting suspension was heated to reflux for 48 h. Then, the methanolic extract was filtered twice through a Whatman filter paper (pore size: 4.0–12 µm) and then concentrated to dryness, yielding the polyphenolic-enriched extract as a paste.

### 2.4. Extraction of Glucosinolate Content

The extraction protocol was performed as previously published [19]. Briefly, one (1.0) gram of the freeze-dried flower watercress sample was mixed with 50 mL of 70% (*v/v*) aqueous methanol. The resulting mixture was heated at 80 °C for 30 min and then it was sonicated for a further 30 min at RT. Afterwards, the resulting suspension was subjected to centrifugation at 3000× *g* for 10 min. The supernatant was diluted with water (1:10) and allowed to stand at room temperature (RT) for 2 h before it was concentrated to dryness. The glucosinolate-enriched fraction was isolated as a viscous oil.

### 2.5. Cell Lines

The human malignant melanoma (A375) cell line was purchased from American Type Culture Collection (ATCC, Manassas, VA, USA), whereas the metastatic malignant melanoma (COLO-679) cell line was purchased from Deutsche Sammlung von Microorganismen und Zellkulturen (DSMZ-Braunschweig, Braunschweig, Germany). The human immortalized keratinocyte (HaCaT) cell line was provided by Dr Sharon Broby (Dermal Toxicology & Effects Group; Centre for Radiation, Chemical and Environmental Hazards; Public Health England, Didcot, UK). Both A375 and HaCaT cell lines were cultured and maintained in DMEM high-glucose media, whereas the COLO-679 cells were kept in RPMI media. All culture mediums were supplemented with serum—10% FBS, amino acids—2 mM *L*-glutamine and antibiotics—1% pen/strep (100 U/mL penicillin, 100 μg/mL streptomycin). Cells were cultured and maintained in a humidified incubator at 37 °C under 5% CO_2_ atmosphere, grown as monolayers and sub-cultured at 80–90% confluency. All cell lines were cultured for 20 passages before new stocks were employed.

### 2.6. Determination of Cytotoxicity

The Alamar Blue assay was utilized. In brief, cells were plated in 100 μL/well into 96-well plates and incubated overnight. The density of A375 was 8000, 4000 and 2000 cells/well whereas that of HaCaT and COLO-679 cells was 10,000, 5000 and 2500 cells/well for 24, 48 and 72 h, respectively. The next day, cells were exposed to a range of concentrations [e.g., 0–2.5% *v/v* (in 0.1% *v/v* DMSO)] over 24–72 h, in the presence or absence of 10 μΜ BAPTA-AM. For control conditions, cells were incubated with complete culture medium only or 0.1% *v/v* DMSO. At the indicated time points, 10 μL of resazurin (1 mg/mL in PBS) was added in each well and maintained for a further 4–6 h at 37 °C under 5% CO_2_. The plates were then subjected to centrifugation, and the absorbance was monitored at 570 nm and 590 nm (reference wavelength) in a microplate reader (LT4500, Labtech, UK). Cell viability was determined and expressed as a percent of control cells.

### 2.7. Transmission Electron Microscope (TEM) Imaging and Quantification

A375 cells at a density of 1.5 × 10^6^ cells/plate were seeded for 24 h followed with exposure to each of the isolated flower watercress extracts or 200 μΜ of *tert*-butyl hydroperoxide for 2, 4 and 24 h. Cells were trypsinized and washed twice with ice-cold PBS. The formed pellets were fixed in 2.5% glutaraldehyde (0.1 M phosphate buffer, pH 7.2) for at least 24 h. Then, fixed cells were washed 3 times with phosphate buffer (0.1 M, pH 7.2) before their post fixation with 1% osmium tetroxide. Afterwards, the fixed samples were dehydrated in graded ethanol (50–100%), cleared in propylene oxide and embedded in an epon/araldite resin mixture. The polymerization occurred at 60 °C for 24 h. Semithin sections of μm thickness were cut using an ultra-microtome (Leica, Reichert UCT, Vienna, Austria), stained with toluidine blue and observed under a light microscope. Gold interference color ultrathin sections (80 nm thickness) were cut and mounted with 300 mesh cooper grids, contrasted with uranyl acetate-zero and lead citrate. The stained grids were examined under a TEM (TALOS L120C, Thermo Scientific, Waltham, MA, USA) operating at 120 kV as described before [40].

### 2.8. Determination of Mitochondrial Membrane Depolarization

A375 cells were seeded overnight on coverslips on a 6-well plate at a density of 300,000 cells/well. The next day, cells were exposed to each of the isolated flower watercress extracts for 2, 4 and 24 h in the presence or absence of 10 μΜ BAPTA-AM. As positive control, cells were treated with 5 μM of carbonyl cyanide *m*-chlorophenyl hydrazine (CCCP) for 30 min. MT-1 dye was diluted in the culture media and cells were further incubated for 30 min at 37 °C under 5% CO_2_ atmosphere. Coverslips were washed twice with complete medium and cells were fixed with 4% *w/v* paraformaldehyde (dissolved in PBS) for 10 min. Upon fixation, coverslips were washed 5× with PBS (5 min/wash). The coverslips were then allowed to dry in air before being mounted on microscope slides. Images were captured via a Zeiss AXIOIMAGER M2 fluorescence microscope utilizing Zeiss Axionvision software (https://www.micro-shop.zeiss.com/en/us/system/software+axiovision-axiovision+program-axiovision+software/10221/), Carl Zeiss Microimaging (Oberkochen, Germany) Finally, the relative fluorescence intensity was determined utilizing the ImageJ (https://imagej.net/ij/) platform [41].

### 2.9. Determination of Caspase Activity

A multiplex assay kit measuring the activity of Caspases-3, -8 and -9 was utilized (Abcam, UK). Briefly, A375 and COLO-679 cells were grown at a density of 8000 and 1000 cells/well, respectively. The following day, cells were exposed to each of the isolated watercress flower extracts in the presence or absence of BAPTA-AM (1 h pre-treatment with 10 μΜ) for 24 h, as previously described [42]. Then, a caspase loading solution was prepared as described in the manufacturer’s protocol. The fluorescence intensity was measured in a fluorescence microplate reader (Synergy H1, Bio-Tek, Winooski, VT, USA) at λ_ex_/λ_em_ = 535/620 nm, 490/525 nm and 370/450 nm for caspase-3, -8 and -9, respectively. Results were expressed as the average relative fluorescence intensity of the corresponding control.

### 2.10. Statistical Analyses

Data were expressed as mean values ± standard deviation (SD). Various comparisons were performed a between control and treated cell groups. Statistical analyses were performed through employing one-way ANOVA with Tukey’s test (for multiple comparisons) using the GraphPad Prism 6 software. Statistical significance was set at *p* < 0.05, <0.01 and <0.001.

## 3. Results

### 3.1. Plant Processing, Extraction and Isolation of Polyphenolics, Glucosinolates and Phenethyl Isothiocyanate-Enriched Fractions

**Figure 1:**
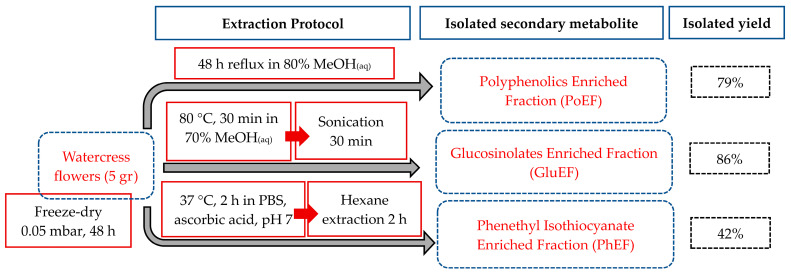
Graphic flowchart of the extraction procedures for the isolation of major secondary metabolites; polyphenols, glucosinolates and phenethyl isothiocyanate from watercress flowers. The extraction yields are shown and calculated based on the isolated mass of each extract.

The experimental pipeline involved the lyophilization and storage of all watercress flower samples. The resultant freeze-dried powder was further processed based on various extraction protocols for the isolation of all the above-mentioned phytochemicals (depending on their chemical properties) as previously described (Figure 1) [18].

### 3.2. Cytotoxic Profile of Isolated Polyphenol, Phenethyl Isothiocyanate and Glucosinolate-Enriched Extracts

**Figure 2:**
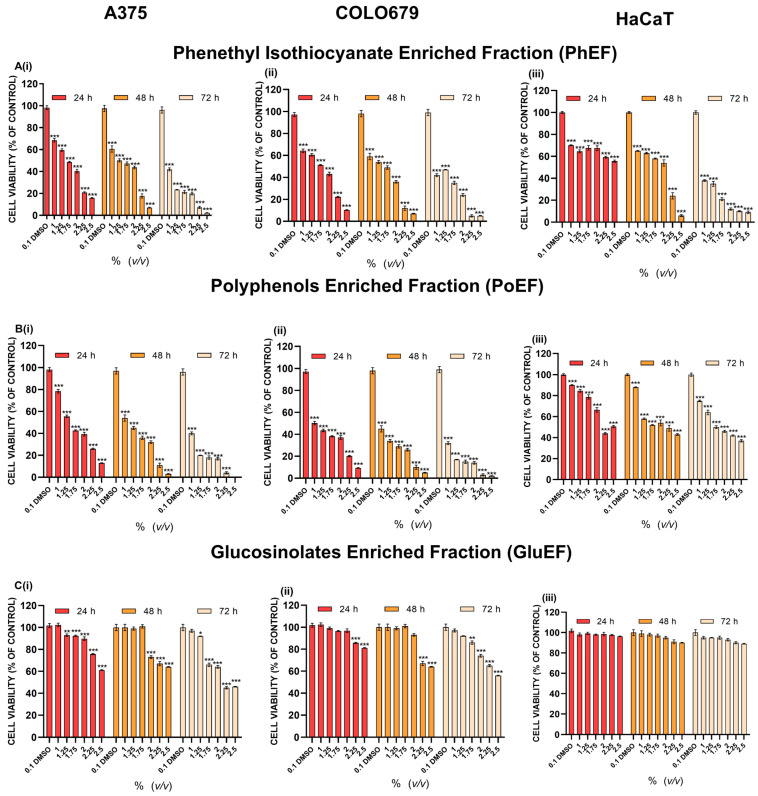
Cytotoxicity profiles of each isolated watercress flower extracts either in phenethyl isothiocyanate (PhEF) (**A**) or polyphenol (PoEF) (**B**) or glucosinolate (GluEF) (**C**) enriched fractions in a cell-based model of human malignant melanoma consisting of (**i**) primary (A375) and (**ii**) metastatic (COLO-679) melanoma as well as (**iii**) non-tumorigenic immortalized keratinocyte (HaCaT) cells exposed to a range of concentrations (1.0–2.5% *v/v*) over 24, 48 and 72 h. Data are expressed as means ± SEM and are representative of three independent experiments. Untreated cells were omitted from the graphical representation. Statistical significance is marked by * at *p* ≤ 0.05, ** at *p* ≤ 0.01 whereas *** *p* < 0.001 relative to corresponding controls (DMSO 0.1% *v/v*).

The isolation and content quantification of each individual extract fraction were performed according to previously published methodologies [19,33]. The cytotoxic potency of each extract fraction was evaluated in an in vitro model of human malignant melanoma consisting of primary (A375) and metastatic (COLO-679) melanoma as well as non-tumorigenic immortalized keratinocyte (HaCaT) cells (Figure 2 and Table 1).

Overall, our results showed that PhEF and PoEF induced cytotoxicity in A375 (Figure 2(Ai,Bi)) and COLO-679 (Figure 2(Aii,Bii)) malignant melanoma cells in a dose- and time- depended manner. Moreover, exposure to these extract fractions also induced cytotoxicity in HaCaT cells, but only after 48 and 72 h of exposure, while at 24 h, the cells remained relatively resistant (Figure 2(Aiii,Biii)). On the other hand, GluEF was the least cytotoxic fraction in both melanoma cell lines (Figure 2(Ci,Cii)) whereas it remained completely unresponsive to HaCaT cells at any time point of exposure (Figure 2(Ciii) and Table 1). Overall, the EC_50_ value of PhEF was shown to be considerably lower in A375 and COLO-679 cells, at each time point of exposure, when compared to those of PoEF and GluEF, thus highlighting its increased cytotoxic potency over the other extract fractions (Table 1).

### 3.3. Characterization of PhEF-Induced Ultrastructural Subcellular Alterations

**Figure 3:**
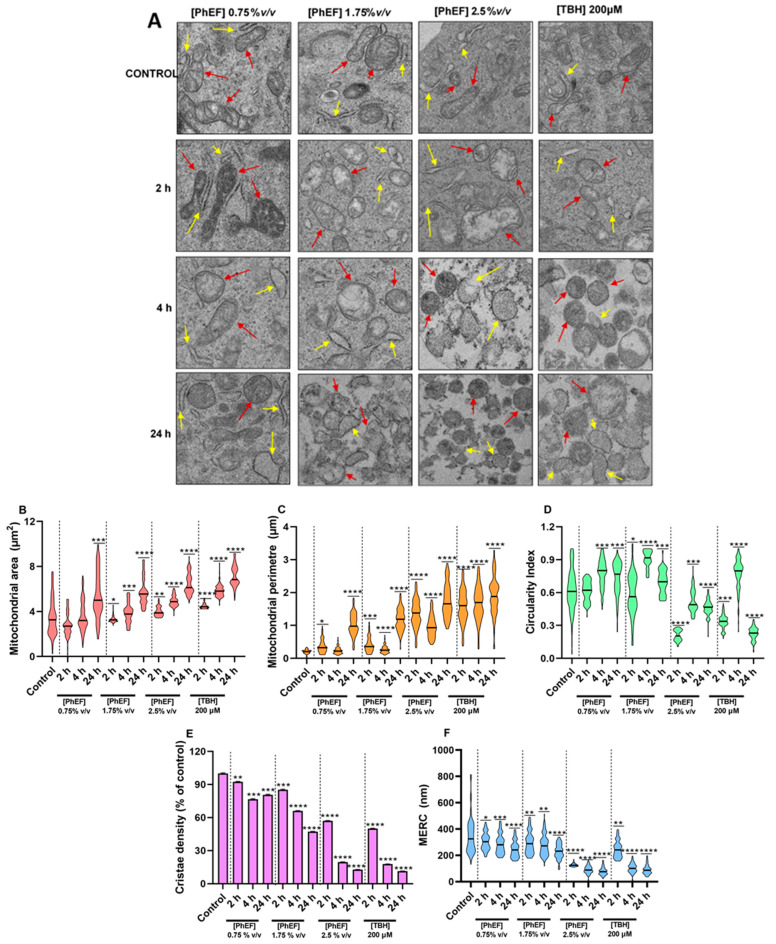
Electron micrographs, along with the corresponding quantitative data, obtained from sections of A375 cells treated with PhEF. Representative electron micrographs obtained from ultra-thin sections (0.1 μm) showing A375 cells treated with various concentrations of PhEF (0–2.5% *v/v*) after 2, 4 and 24 h of exposure. Red and yellow arrows indicate mitochondria and endoplasmic reticulum, respectively (**A**); quantification of mitochondrial area (μm^2^) (**B**); mitochondria perimeter (μm) (**C**); mitochondrial circularity index (**D**); cristae density (% of control) (**E**); mitochondria–ER contact (MERC) site length (nm) (**F**). Data are expressed as means ± SEM; statistical significance is indicated by * *p* < 0.05, ** *p* < 0.01, *** *p* < 0.001 and **** *p* < 0.0001 relative to corresponding controls.

Transmission electron microscopy (TEM) was utilized to detect “early” subcellular markers associated with apoptotic induction through determining ultrastructural alterations including those of mitochondrial area, perimeter, circularity index and cristae density as well as mitochondria–endoplasmic reticulum contacts (MERCs). For this reason, A375 cells were exposed to concentrations either (i) below EC_50_, 0.75% *v/v* PhEF (as it associates with cell viability levels close to control ones), (ii) at EC_50_, 1.75% *v/v* PhEF (as it reduces cell viability levels to 50% of control), or (iii) higher than EC_50_, 2.5% *v/v* PhEF (as it considerably reduces cell viability levels to about 80%), over 2, 4 and 24 h. Furthermore, *tert*-butyl hydroperoxide (TBH; 200 μM) was utilized as a positive control. Electron micrographs were collected at each time point of exposure (Figure 3A), and all the parameters associated with the above-mentioned ultrastructural alterations were quantified via ImageJ software as previously described [43] (Figure 3B–F and Table 2).

Specifically, there was a marked increase in mitochondrial area and perimeter which was shown to be both time- and concentration-dependent. This effect was intensified as time and concentration of exposure was increased (Figure 3B,C and Table 2). On the other hand, the circularity index was decreased only at the lethal concentration of PhEF, whereas cristae density was decreased under all experimental conditions but of a different magnitude, with the effect being intensified over prolonged duration and concentration of exposure conditions (Figure 3D,E and Table 2). Last, the distance between mitochondria and endoplasmic reticulum contacts (MERCs) was shown to be shorter but only at lethal concentrations of PhEF, regardless of exposure time (Figure 3F and Table 2).

### 3.4. Determination of PhEF-Induced Mitochondrial Membrane Depolarization

**Figure 4:**
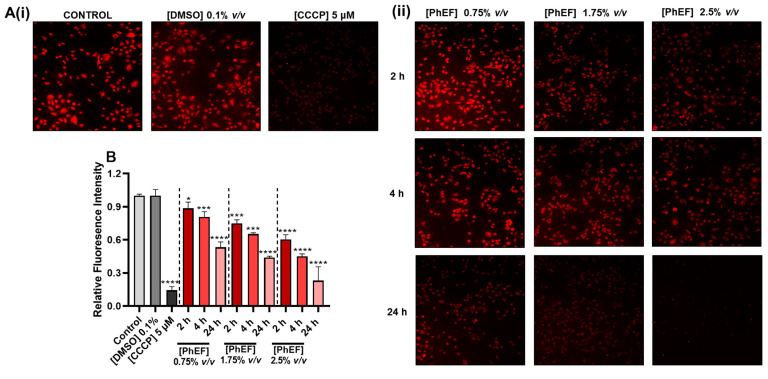
Ability of PhEF to induce mitochondrial membrane depolarization (ΔΨ*_m_*) on fixed A375 cells treated with a range of concentrations of PhEF (0–2.5% *v/v*) over 2, 4 and 24 h utilizing a Mito-MP fluorescent dye **A**(**ii**). The quantification of ΔΨ*_m_* was proportional to the fluorescent intensity of the MT-1 fluorescent dye at λ_ex_ = 530 nm/λ_em_ = 570 nm (**B**). CCCP (5 μΜ) and DMSO (0.1% *v/v*) were used as positive and negative controls, respectively **A**(**i**). Data shown are means ± SEM and are representative of three independent experiments; statistical significance is indicated by * *p ≤* 0.05 *** *p* < 0.001 and **** *p* < 0.0001 relative to corresponding controls.

In this set of experiments, we sought to investigate the potential of different concentrations of PhEF to induce mitochondrial membrane depolarization (ΔΨ*_m_*) utilizing fluorescence microscopy through the use of a commercially available MT-1 dye under control and CCCP (a compound inducing mitochondrial membrane depolarization) experimental conditions (Figure 4(Ai)).

At non-lethal concentrations of PhEF (0.75% *v/v*), we observed that the mitochondrial membrane potential remained intact after 2–4 h of exposure but not after 24 h, when there was significant depolarization. On the contrary, under sub-lethal (1.75% *v/v*) and lethal (2.5% *v/v*) concentrations of PhEF, significant depolarization was observed as early as 2–4 h after exposure, an effect which was significantly potentiated after 24 h (Figure 4(Aii,B)).

### 3.5. Determination of PhEF-Induced Ca^2+^ Efflux Perturbations

**Figure 5:**
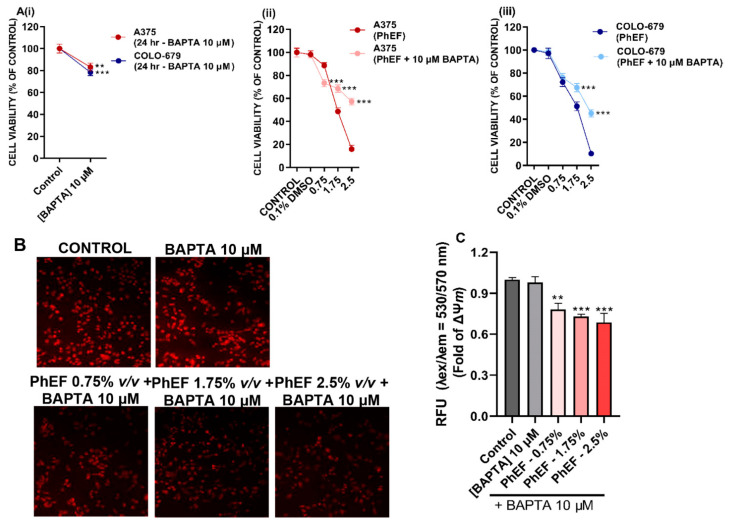
Cytotoxic profile of BAPTA (10 μΜ) upon exposure of A375 and COLO-679 cells, for 24 h. Panel (**Ai**); Co-treatment with BAPTA and PhEF prevents reduction of viability in A375 (**Aii**) and COLO-679 (**Aiii**) cells as well as depolarization of mitochondrial membranes (**B**), an effect which was quantified as fold of ΔΨ*m* (**C**). Data shown are means ± SEM and are representative of three independent experiments; statistical significance is indicated by ** at *p* ≤0.01 and *** at *p* < 0.001 relative to corresponding controls.

Next, we sought to determine perturbations in Ca^2+^ efflux homeostasis for which a Ca^2+^ chelator, BAPTA-AM, was employed. Both A375 and COLO-679 cells were treated with BAPTA-AM, and cell viability levels were shown to be slightly reduced after 24 h of exposure (Figure 5(Ai)). Moreover, our data showed that the reduction in cell viability upon treatment with BAPTA-AM (at different concentrations of PhEF) was significantly less than without BAPTA-AM co-treatment in both A375 (Figure 5(Aii)) and COLO-679 (Figure 5(Aiii)) cells, suggesting the involvement of Ca^2+^ in PhEF-induced cytotoxicity.

In another set of experiments, our data revealed that co-treatment with BAPTA-AM minimized mitochondrial membrane depolarization observed at different concentrations of PhEF over 24 h of exposure (Figure 5B,C), once again suggesting the role of Ca^2+^ efflux in mitochondrial membrane depolarization.

### 3.6. Determination of PhEF-Induced Activation of Caspases-8, -9 and -3

**Figure 6:**
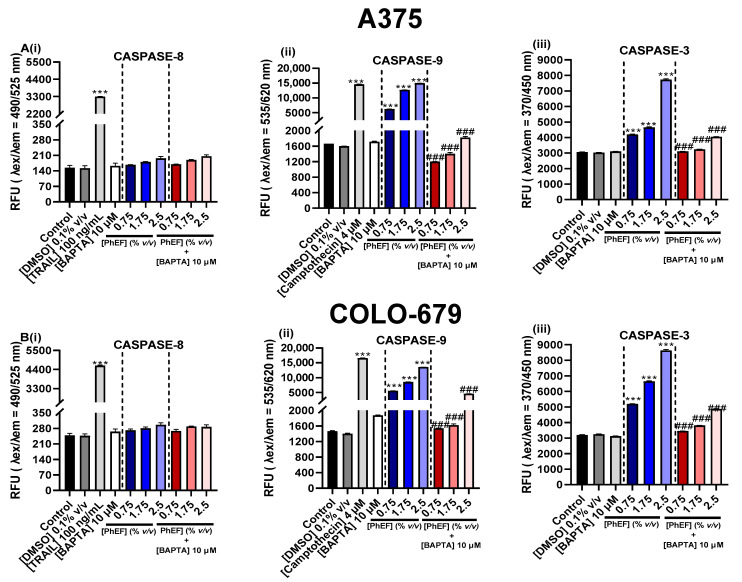
The effect of PhEF in activating Caspases-8 (**Ai**,**Bi**), -9 (**Aii**,**Bii**) and -3 (**Aiii**,**Biii**) was determined in A375 and COLO-679 cells following exposure to PhEF (0.75–2.5% *v/v*) in the presence of 10 μM BAPTA for 24 h. TRAIL (100 ng/mL) and Camptothesin (4 μM) were utilized as positive controls for extrinsic and intrinsic apoptosis, respectively. Data shown are means ± SEM and are representative of three independent experiments; *** denote statistical significance when compared to their respective control (DMSO 0.1% *v/v*) at *p* < 0.001, whereas ^###^ denote statistical significance at *p* < 0.001 when compared to cells treated with PhEF only.

Under exposure conditions of 0–2.5% *v/v* over 24 h, PhEF was shown to induce the activation of apoptosis, measured as the activity levels of Caspases-3, -8 and -9, utilizing a commercially available multiplex assay kit (Figure 6). Specifically, our data revealed that exposure to different concentrations of PhEF (0.75–2.5% *v/v*) led to an increase in the activity levels of Caspases-9 (Figure 6(Aii,Bii)) and -3 (Figure 6(Aiii,Biii)), while caspase-8 remained unresponsive (Figure 6(Ai,Bi)), a pattern which was similar between A375 (Figure 6(Ai–Aiii)) and COLO-679 (Figure 6(Bi–Biii)) malignant melanoma cells.

In addition, it was demonstrated that the activation of caspases-9 and -3 was increased in a PhEF concentration-dependent manner. Finally, the inhibition of Ca^2+^ efflux, by BAPTA-AM, inhibited the activation of apoptosis (by means of caspases-9 and -3 activity levels), suggesting its potential role in modulating the apoptotic response. Once again, this response pattern was similar between A375 and COLO-679 cells.

## 4. Discussion

It has been previously documented that among the aerial parts of watercress, flowers possess the highest concentration of ITCs, polyphenols and glucosinolates [19,44]. This study aimed to evaluate the anticancer capacity of various isolated watercress flower extracts enriched in either PEITC (PhEF) polyphenols (PoEF) or glucosinolates (GluEF). Our findings revealed a significant reduction in cell viability in A375 and COLO-679 malignant melanoma cells exposed to either PhEF or PoEF, with the latter one being the least cytotoxic among the two extract fractions. Moreover, our data demonstrated that the toxicity induced by each of these extracts affected non-tumorigenic keratinocytes (HaCaT) at 48 h of exposure onwards. In previous studies [45,46,47,48], it was documented that malignant melanoma cells exert a dysregulated redox state, as there are higher basal levels of ROS production compared to those of keratinocytes and/or fibroblasts. This is because elevated ROS levels are essential for melanoma cancer transformation and progression, whereas ROS scavenging appears to suppress their metastatic capacity [48,49,50,51,52,53]. This might provide a rationale for the observed PoEF-induced reduction in cell viability levels, as it consists of various polyphenol compounds (known for their ROS-scavenging antioxidant properties), thus affecting ROS homeostasis in malignant melanoma cells. Moreover, the biological activity of the glucosinolates-enriched extract fraction (GluEF) was examined. Our results demonstrated a reduction in cell viability levels only at the experimental conditions of high extract concentrations and/or prolonged time periods of exposure. Our data are in agreement with the work of others indicating that intact glucosinolates are not capable of inducing cytotoxicity against various experimental cancer models [54,55,56,57,58]. On the contrary, the addition of myrosinase can promote their hydrolysis and thus can yield cytotoxic by-products including nitriles, thiocyanates, ITCs, epithionitriles and 1,3-oxazolidine-2-thiones [33,54,59,60].

Several cytotoxic mechanisms have been previously proposed regarding PEITC-induced cytotoxicity, including ROS generation, cell cycle growth arrest [61,62,63] and apoptosis [18,64,65,66]. In the current study, various approaches were employed to monitor “early” and “late” apoptotic stages at various time points of exposure and under different concentrations of PhEF (non-lethal, sub-lethal and lethal). To these ends, our data have revealed ultrastructural alterations associated with hallmarks of apoptotic induction including mitochondrial area, perimeter and circularity index, as well as cristae density and mitochondria–ER contact sites. As previously suggested, apoptotic induction causes sequential mitochondrial structural alterations which are classified as normal-vesicular, vesicular-swollen and swollen [67]. During our study, we were able to observe these morphological changes as normal-vesicular and swollen over 4 h and 24 h post exposure, respectively. More specifically, mitochondria swelling is characterized by the opening of the mitochondria permeability transition pore (mPTP), which leads to the dilution of the inner matrix (appearing as less electron-dense regions under TEM) and an increase in the overall size of mitochondria (quantitated as mitochondrial area and perimeter) [68,69,70]. Moreover, the observed mitochondrial remodeling was also reflected in the cristae morphology, as shown from the electron micrographs. Specifically, our data show the displacement of the cristae to the periphery, with the structure being strongly affected by the severity of the exposure conditions as they appeared to be disoriented, shorter and fewer in number, thereby potentially leading to cristae vesiculation, a process preceding mitochondria swelling which facilitates the diffusion of cytochrome-c, from the intracristal compartments, upon mitochondrial membrane rupture [71,72,73,74,75]. Finally, we have shown that the mitochondria remodeling at the early time points of PhEF exposure (2–4 h) was not accompanied by mitochondrial membrane depolarization; however, this was not the case with the later time points of exposure (24 h), concomitant with mitochondrial swelling and the complete loss of cristae. At these exposure conditions (24 h), we were able to observe the loss of mitochondrial membrane potential and also that the effect was intensified in a PhEF concentration-dependent manner.

Last, we observed that exposure to PhEF led to ER stress (indicated as swollen ER), often associated with Ca^2+^ overload. In previous studies, it was demonstrated that PEITC can induce Ca^2+^ efflux from the ER to the cytosol and to the mitochondria, thus promoting cell death in an in vitro model of cholangiocarcinoma [31]. The mobilization of Ca^2+^ from the ER to mitochondria generates an osmotic force (via water absorption from the cytoplasm) which eventually dilates the ER [76,77]. It has also been suggested that Ca^2+^ is a mediator between the ER and mitochondria, and so it acts as a crucial factor in apoptotic activation. In fact, Ca^2+^ can induce mitochondrial swelling upon various apoptotic stimuli, thereby causing membrane rupture and the release of apoptotic factors (e.g., pro-caspase-9, cytochrome-c, Smac/DIABLO and endonuclease G) from the intramembranous space to the cytosol [78,79,80,81,82]. Furthermore, it has been demonstrated that the proapoptotic members of the Bcl-2 family (e.g., BAK, BAX) can modulate apoptotic induction through Ca^2+^ mobilization from the ER to mitochondria [83,84,85,86]. Consequently, inhibiting Ca^2+^ efflux (using BAPTA-AM) resulted in restoring the malignant phenotype of A375 and COLO-679 malignant melanoma cells through inhibiting the activation of initiator caspase-9 and effector caspase-3, thereby highlighting the role of Ca^2+^ efflux as a key intracellular mediator in signaling the PhEF-induced activation of intrinsic apoptosis.

Limitations of the current study include the validation of current findings in an in vivo experimental model. To this end, our current preliminary results have already led us to the design of an experimental set up which will allow us to validate our results in an established in vivo model in the future. Additionally, the current data needs to further document its therapeutic relevance in adjuvant combinatorial protocols through utilizing specific isolated extract fractions in combination with clinically relevant drugs used for the therapeutic management of malignant melanoma patients.

Finally, the advantage/significance of the current study is that it allows us to determine the role of a naturally derived extract as an adjuvant in the development of novel combinatorial treatment options for the clinical management of malignant melanoma patients, primarily by means of minimizing the side toxicity issues often associated with the use of clinical drugs.

## 5. Conclusions

Overall, we have demonstrated that a watercress flower-based PEITC-enriched extract fraction (PhEF) induces significant cytotoxicity against primary and metastatic malignant melanoma cells, while non-tumorigenic keratinocytes remain relatively unaffected. In addition, we were able to observe TEM-based subcellular ultrastructural changes which can potentially act as “early” markers of apoptotic induction, including mitochondrial area and perimeter as well as a decrease in mitochondrial cristae and density. Subsequently, such changes can contribute to mitochondrial swelling, a well-characterized process of apoptotic induction. On the other hand, the observed mitochondrial membrane depolarization (a well-established “late” apoptotic marker), together with perturbations in Ca^2+^ homeostasis, led to PhEF-induced activation of the intrinsic apoptotic cascade.

## Figures and Tables

**Table 1:** EC_50_ values (expressed as % *v*/*v*) for each watercress flower extract fraction. EC_50_ were estimated in all cell lines, at each time point of exposure, through utilizing an online EC_50_ calculator platform (Very Simple IC_50_ Tool Kit, available online: http://www.ic50.tk/ (accessed on 24 November 2022). n.d. represents data not determined.

EC_50_ (% *v/v*)									
	PhEF			PoEF			GluEF		
Time(h)	A375	COLO-679	HaCaT	A375	COLO-679	HaCaT	A375	COLO-679	HaCaT
24	1.70 ± 0.10	1.80 ± 0.40	n.d.	1.8 ± 0.02	2.00 ± 0.10	n.d.	n.d.	n.d.	n.d.
48	1.25 ± 0.01	1.20 ± 0.20	2.10 ± 0.20	1.6 ± 0.20	1.30 ± 0.01	1.80 ± 0.10	n.d.	n.d.	n.d.
72	1.05 ± 0.01	1.00 ± 0.10	2.00 ± 0.10	1.1 ± 0.20	1.10 ± 0.02	1.20 ± 0.20	2.20 ± 0.02	n.d.	n.d.

**Table 2:** Fold change of ultrastructural parameters including mitochondrial area, perimeter, circularity index and mitochondria–ER contacts upon exposure of A375 cells to various concentrations of PhEF (0.75, 1.75 and 2.5% *v/v*), at 2, 4 and 24 h.

Fold of Change (Relative to Control)													
		[PhEF] 0.75% *v/v*			[PhEF] 1.75% *v/v*			[PhEF] 2.50% *v/v*			[TBH] 200 μM		
	[DMSO] 0.1% *v/v*	2 h	4 h	24 h	2 h	4 h	24 h	2 h	4 h	24 h	2 h	4 h	24 h
**Mitochondrial** **area (μm^2^)**	1	0.8 ± 0.10	1.00 ± 0.10	1.50 ± 0.20	0.90 ± 0.02	1.10 ± 0.08	1.60 ± 0.10	1.10 ± 0.04	1.40 ± 0.04	1.80 ± 0.08	1.3 ± 0.02	1.70 ± 0.05	2.06 ± 0.08
**Mitochondrial** **perimeter (μm)**	1	1.77 ± 0.02	1.15 ± 0.008	5.00 ± 0.03	1.97 ± 0.02	1.30 ± 0.01	5.70 ± 0.03	6.59 ± 0.04	4.72 ± 0.03	8.52 ± 0.04	7.6 ± 0.04	8.06 ± 0.04	9.23 ± 0.04
**Cristae density** **(% control)**	1	0.92 ± 0.06	0.77 ± 0.02	0.81 ± 0.03	0.85 ± 0.03	0.66 ± 0.02	0.47 ± 0.02	0.57 ± 0.02	0.19 ± 0.001	0.13 ± 0.01	0.50 ± 0.02	0.18 ± 0.001	0.12 ± 0.001
**Circularity** **Index**	1	1.00 ± 0.09	1.28 ± 0.11	1.17 ± 0.11	0.94 ± 0.07	1.45 ± 0.08	1.12 ± 0.04	0.34 ± 0.02	0.83 ± 0.05	0.74 ± 0.04	0.54 ± 0.02	1.24 ± 0.10	0.36 ± 0.002
**MERC length** **(nm)**	1	0.88 ± 0.06	0.82 ± 0.04	0.71 ± 0.05	0.85 ± 0.04	0.80 ± 0.02	0.66 ± 0.02	0.36 ± 0.02	0.26 ± 0.01	0.23 ± 0.002	0.71 ± 0.07	0.28 ± 0.01	0.26 ± 0.02

## Data Availability

The datasets generated during the current study are available from the corresponding author on reasonable request.
